# Emergence of a novel PRRSV-1 strain in mainland China: A recombinant strain derived from the two commercial modified live viruses Amervac and DV

**DOI:** 10.3389/fvets.2022.974743

**Published:** 2022-09-09

**Authors:** Qi Sun, Hu Xu, Chao Li, Bangjun Gong, Zhen Li, Zhi-Jun Tian, Hongliang Zhang

**Affiliations:** ^1^State Key Laboratory of Veterinary Biotechnology, Harbin Veterinary Research Institute, Chinese Academy of Agricultural Sciences, Harbin, China; ^2^Pingdingshan Center for Animal Disease Control and Prevention, Pingdingshan, China

**Keywords:** PRRSV-1, recombination, novel strains, vaccine, DV, Amervac

## Abstract

Porcine reproductive and respiratory syndrome virus 1 (PRRSV-1) is one of the main pathogens causing porcine reproductive and respiratory syndrome (PRRS). In recent years, the rate of PRRSV-1 detection in China has gradually increased, and the PRRSV-1 strains reported in China belong to subtype I (Global; Clade A-L). In the present study, a novel PRRSV-1 strain, TZJ2134, was found during epidemiological surveillance of PRRSV-1 in Shandong Province in China. We obtained two fragments of the TZJ2134 genome: TZJ2134-L12 (located at nt 1672-nt 2112 in the partial Nsp2 gene) and TZJ2134-(A+B) (located at nt 7463-nt 11272 in the partial Nsp9, complete Nsp10 and partial Nsp11 genes). Phylogenetic and recombination analyses based on the two sequences showed that TZJ2134 is a recombinant strain derived from two commercial PRRSV-1 modified live vaccine (MLV) strains (the Amervac vaccine and DV vaccine strains) that formed a new recombinant subgroup of DV+Amervac-like isolates with other strains. However, PRRSV-1 MLV is not currently allowed for use in China. This study is the first to detected recombinant PRRSV-1 MLV strain in China and provides new data for the epidemiological study of PRRSV-1 in China. The existence of the TZJ2134 strain is a reminder that the swine surveillance at the Chinese customs should be strengthened.

## Introduction

Porcine reproductive and respiratory syndrome (PRRS) is a highly contagious disease causing substantial economic losses in the swine industry worldwide. It is mainly characterized by widespread reproductive failure in pregnant sows and respiratory symptoms in pigs of all ages (1). PRRSV is divided into two species, namely, Betaarterivirus suid 1 (PRRSV-1) and Betaarterivirus suid 2 (PRRSV-2) (https://talk.ictvonline.org/taxonomy/p/taxonomy-history?taxnode_id=20171832), which share only 50–60% nucleotide sequence identity (2) and have attracted increasing attention due to the high incidence of PRRSV mutation and recombination. Based on the phylogenetic analysis of ORF5 nucleotide sequences and the global PRRSV classification system, PRRSV-1 is divided into four subtypes: subtype I (Global; Clade A-L), subtype I (Russian), subtype II, and subtype III. PRRSV-2 can be divided into nine subtypes: lineages 1~9 (3).

PRRSV-1 and PRRSV-2 have significant differences in geographical distribution. PRRSV-2 was isolated in 1992 in America (ATCC-VR2332, the North American prototypic strain) (4) and is mainly prevalent in North America and Asia (5). PRRSV-1 was isolated in 1991 in the Netherlands (Lelystad virus, the European prototypic strain) (6) and is mainly prevalent in Europe. However, only subtype I (Global; Clade A-L) has spread to continents other than Europe. The remaining subtypes have been reported only in Eastern European countries and Russia (7). In 1997, the isolated PRRSV-1 strain B13 (GenBank: AY633973) was first detected by customs in Mainland China. In 2011, Chen et al. isolated PRRSV-1 strains (BJEU06-1, NMEU09-1) in China, which was the first report of wild PRRSV-1 isolates on a pig farm in mainland China (8). To date, PRRSV-1 has been prevalent in at least 20 provinces in China (9–13). In recent years, the rate of PRRSV-1 detection in China has gradually increased. The PRRSV-1 strains reported in China all belong to subtype I (Global) and can be divided into four subgroups (NMEU09-1-like, Amervac-like, HKEU16-like, and BJEU06-1-like isolates) (14). However, the number of PRRSV-1 infections detected in China was found to be lower than that of PRRSV-2 infections (15, 16). We speculated that the reason might be that PRRSV-1 is not given much attention because it is not the main epidemic strain in China, and there is evidence that most PRRSV-1-infected pigs in China exhibit mild clinical symptoms (10, 17). Even so, there is still a risk of the recombination of vaccine strains to create new ones, so the effect of PRRSV-1 on pigs should not be ignored.

Vaccination is a key strategy for PRRSV-1 prevention and control. In the late 1990s, PRRSV-1 MLV was used in Europe (18). Nevertheless, the particularly high variability of PRRSV and the possibility of vaccine revertant PRRSV emerging in pigs vaccinated with PRRSV MLV could result in recombination between different MLV strains (19, 20) or recombination between MLV strains and wild-type PRRSV strains (14, 21, 22). This potential indicates the importance of the rational use of vaccines. In this study, a novel PRRSV-1 strain was identified in an epidemiological investigation of PRRSV in China; this strain was derived from the recombination of two commercial PRRSV-1 MLV strains (Amervac vaccine strain and DV vaccine strain).

## Materials and methods

In 2021, a clinical (lung) sample, TZJ2134, was detected in an aborted sow from a very small backyard farm in Shandong Province of China that did not utilize vaccination. Tissue sample processing, RNA extraction, cDNA preparation, RT–PCR and genome sequencing were performed as described previously (23, 24). The TZJ2134 strain was identified as PRRSV-1 by RT–PCR with the primer L12 (primer for specific detection of PRRSV-1) (Supplementary Table S1). Based on the complete genomic sequence of PRRSV-1, eight primer pairs were designed for RT–PCR amplification and sequencing (Supplementary Table S1), and two overlapping fragments of TZJ2134 were amplified by RT–PCR. Each PCR product was purified with the E.Z.N.A.® Gel Extraction Kit-Omega Bio-Tek, cloned into the pMD18-T vector according to the manufacturer's instructions, and then submitted to Comate Bioscience Co., Ltd. (Changchun, China) for sequencing.

Sequence analysis was performed with DNASTAR (version 7.1) software. The resulting sequences were assembled using SeqMan. Phylogenetic trees were generated and molecular evolutionary analyses were performed by using the neighbor-joining method in MEGA 7.0. with 1,000 bootstrap replicates for each node (25). The generated phylogenetic tree was annotated using iTOL online software (https://itol.embl.de/) (26). Sixty-five representative PRRSV-1 strains from different subtypes by referring to the following articles (9, 11–14, 27) available in GenBank were used for comparative sequence analyses in this study.

To analyze the recombination events, RDP4 and the NCBI BLAST results were used to identify the major and minor parental strains (28). Seven different algorithms (RDP, GeneConv, BootScan, MaxChi, Chimera, SiScan, and 3 Seq) embedded in the RDP4 software package with Bonferroni correction were utilized to detect recombination events and breakpoints. Detection using four or more of the seven methods implemented in RDP4 was taken as significant evidence for recombination. Recombination events were further confirmed by SimPlot 3.5.1 (29), and boot scanning analysis was performed with a 200-bp window, sliding along the genome alignment with a step size of 20 bp. Other strains in the same group as TZJ2134-(A+B) (PRRS-FR-2014-56-11-1, DK-2011-05-23-9 and OLot/91 strain) were analyzed by SimPlot 3.5.1 (29) using the same method.

## Results and discussion

**Figure 1:**
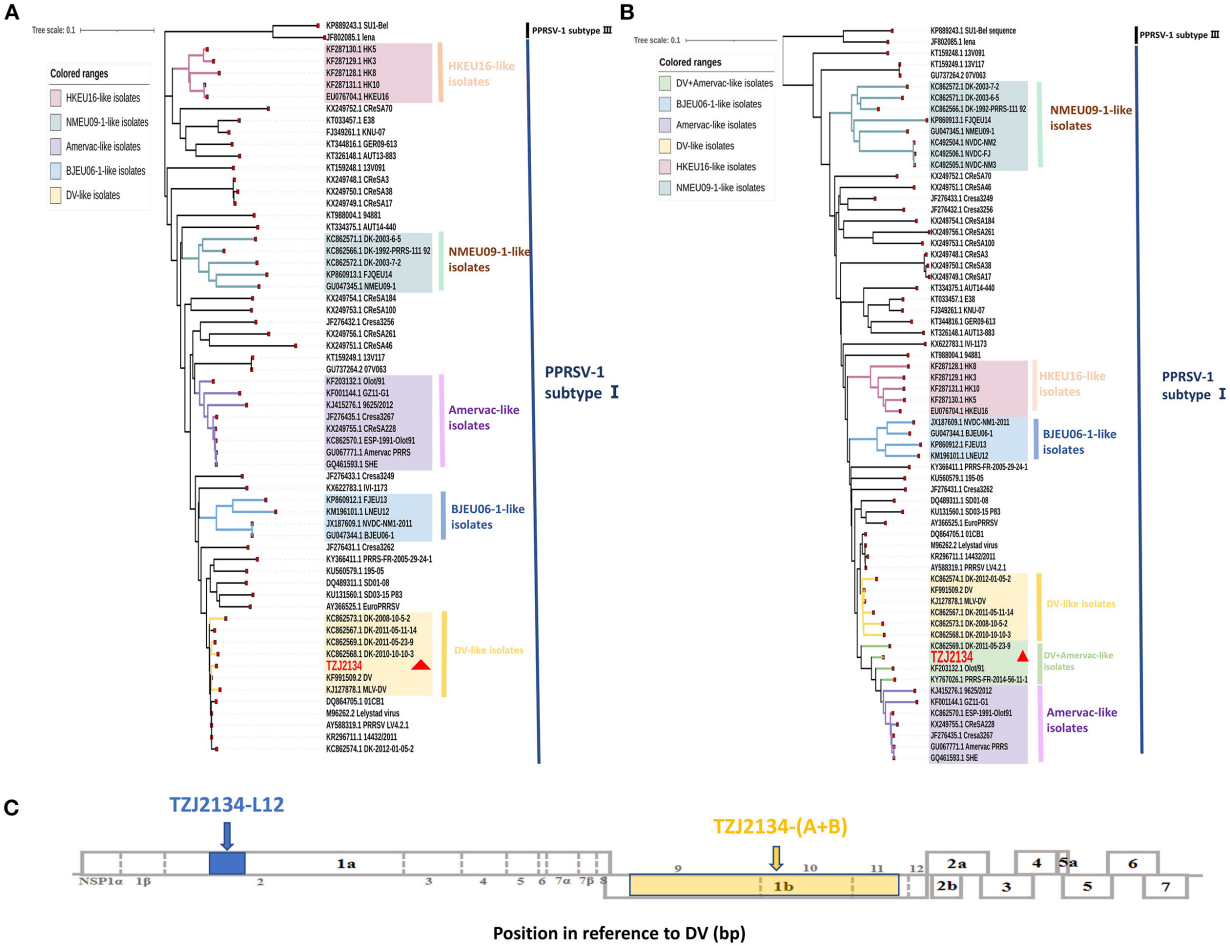
Phylogenetic analysis based on the TZJ2134-L12 and TZJ2134-(A+B) gene sequences and positions of TZJ2134-L12 and TZJ2134-(A+B) in the whole genome structure. **(A)** Phylogenetic tree based on TZJ2134-L12 of 60 PRRSV-1 strains. The TZJ2134 and DV strains belong to DV-like isolates, which are shown in yellow. **(B)** Phylogenetic tree based on TZJ2134-(A+B) of 65 PRRSV-1 strains. A novel subgroup DV+Amervac-like isolate is shown in green. **(C)** The whole genome structure of PRRSV, which shows the positions of 10 open reading frames. TZJ2134-L12 is located at nt 1672-nt 2112 in partial Nsp2, shown in blue; TZJ2134-(A+B) is located at nt 7463-nt 11272 in partial Nsp9, complete Nsp10 and partial Nsp11, shown in yellow. TZJ2134 is indicated with a red triangle in phylogenetic trees.

In 2021, the TZJ2134 strain was isolated in Shandong Province in China and showed positivity for PRRSV-1 by detection using primer L12 (Supplementary Table S1). Subsequently, a sequence of TZJ2134 (TZJ2134-L12) was obtained by amplification with the detection primer L12, located in the partial Nsp2 gene (nt 1672-nt 2112 of DV) (Figure 1C). Phylogenetic analysis showed that all Chinese PRRSV-1 isolates belonged to subtype I and could be divided into four subgroups (Amervac-like, BJEU06-1-like, HKEU16-like, and NMEU09-1-like isolates) (Figure 1). TZJ2134-L12 belonged to DV-like isolates and shared the highest sequence identity (99.54%) with the DV vaccine strain (Table 1).

**Table 1:** Nucleotide similarity between different gene fragments of TZJ2134 and vaccine strains of PRRSV-1.

**Different gene**	**Reference vaccine**	
**fragments of TZJ2134**	**strains of PRRSV-1**	
	**DV**	**Amervac_PRRS**
TZJ2134-L12	99.3%	93.0%
TZJ2134-(A+B)	97.1%	97.4%
TZJ2134-A	99.5%	95.2%
TZJ2134-B	94.5%	99.7%

To obtain the complete genome of TZJ2134, we designed eight pairs of primers for amplification. Unfortunately, the whole-genome sequence could not be obtained after repeated attempts, and only two overlapping fragments of the TZJ2134 genome were amplified by using the primers Ly-E and Ly-F (Supplementary Table S1). Our primers have amplified multiple PRRSV-1 whole genome sequences in other studies (30). Lung sample was kept frozen during transport, and we designed multiple pairs of primers for amplification using the obtained TZJ2134 sequence, but no more PRRSV-1 nucleotide sequences were obtained in the end. We speculate that there may be three reasons. Firstly, TZJ2134 may have a low viral load in the host. Secondly, although we received a frozen sample, but it is not clear how the samples were stored before transport, which may also contribute to the degradation of viral nucleic acid. Thirdly, there may be high level structure (such as hairpin structure) in some parts of the genome after virus recombination, which leads to the inability or low binding efficiency of primers. Therefore, we did not obtain the full genome sequence of TZJ2134. Subsequently, the resulting sequences of the two overlapping fragments were assembled into a contig [named TZJ2134-(A+B)]. Further genetic evolution and homology analyses showed that TZJ2134-(A+B) located at nt 7463-nt 11272 (3810 nt in length) in partial Nsp9, complete Nsp10 and partial Nsp11 (Figure 1C) shared 97.1% nucleotide identity with the DV vaccine strain and 97.4% nucleotide identity with the Amervac vaccine strain (Table 1). To establish a genetic relationship between TZJ2134-(A+B) and other PRRSV-1 isolates, we generated a phylogenetic tree based on 65 PRRSV-1 strains in GenBank that are prevalent in China and the world (Supplementary Table S2). Phylogenetic analysis showed that TZJ2134-(A+B) was an intermediate between Amervac-like isolates and DV-like isolates and formed a separate subgroup (DV+Amervac-like isolates) with PRRS-FR-2014-56-11-1, DK-2011-05-23-9 and OLot/91 strains (Figure 1B).

**Figure 2:**
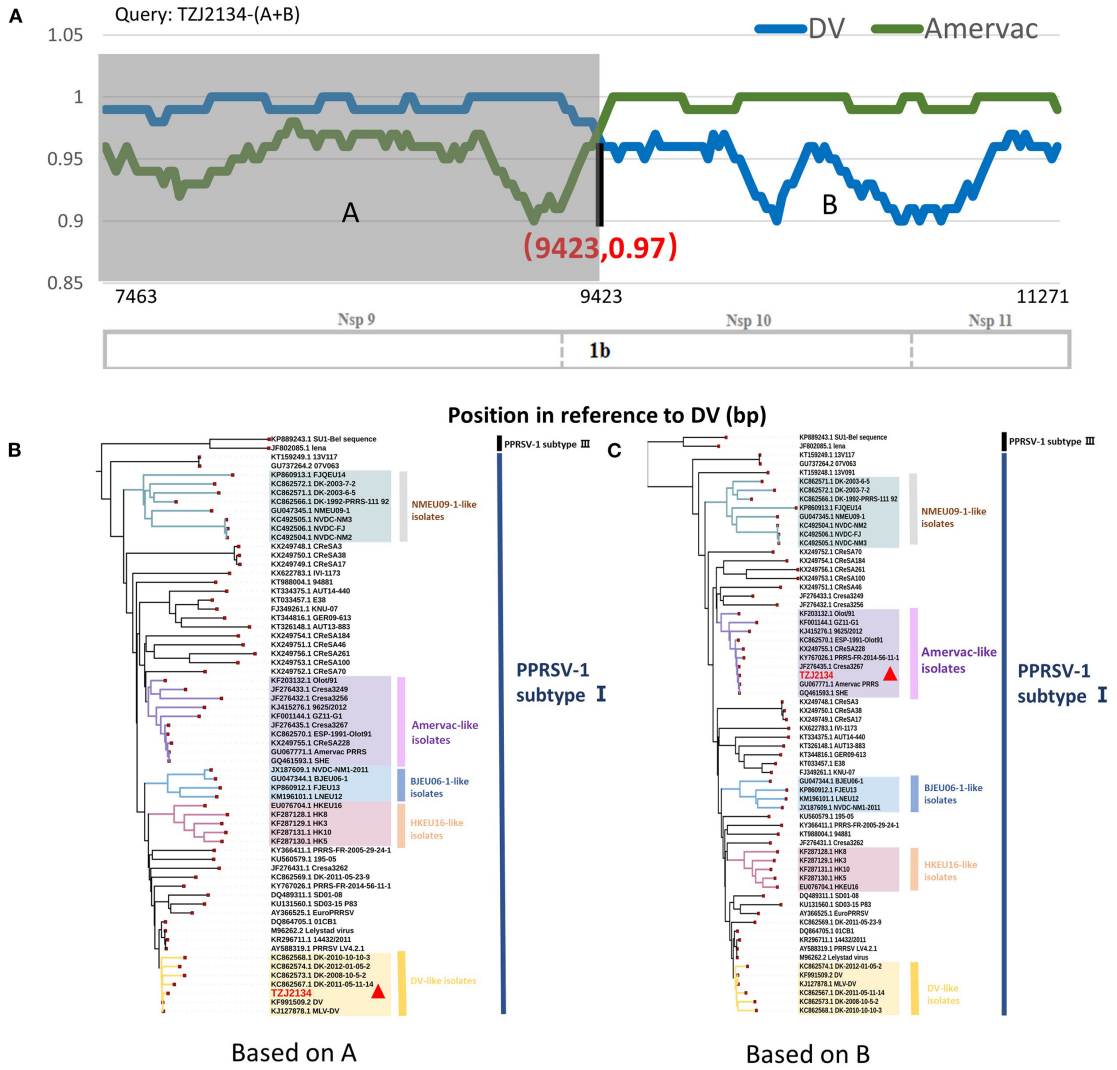
Recombination analysis of TZJ2134-(A+B) and phylogenetic analysis of TZJ2134-A and TZJ2134-B. **(A)** Recombination analysis of TZJ2134-(A+B). Comparison was performed using TZJ2134-(A+B) as the query sequence and DV (blue) and Amervac (green) as the parent strains. The background color of the DV parental region is gray, whereas that of the Amervac parental region is white. Below the similarity plots is the partial genome structure of PRRSV, which shows the positions of three nonstructural proteins (Nsp9, Nsp10, and Nsp11). **(B)** Phylogenetic analysis of TZJ2134-A. The parental group (DV-like isolates) is shown in yellow. **(C)** Phylogenetic analysis of TZJ2134-B. The parental group (Amervac-like isolates) is shown in purple, and the query strain TZJ2134 is indicated with a red triangle.

BLAST analysis showed that the 5' end and 3' end of the TZJ2134-(A+B) sequence had high homology with DV and Amervac strains, respectively (Data not shown). RDP4 and SimPlot (version 3.5.1) were used to test for recombination of TZJ2134-(A+B). The RDP4 analysis results showed that TZJ2134-(A+B) was a recombinant strain from Amervac and DV vaccine strains with a potential crossover event spanning Nsp10. Additionally, the recombination event was further confirmed by SimPlot 3.5.1, which showed that the recombination breakpoint was located in Nsp10 (nt 9423) (Figure 2A). The recombination break point is not located at the splicing of the two nucleotide sequences, thus TZJ2134 is a natural recombinant virus. Based on the putative recombination breakpoint (nt 9243), we divided TZJ2134-(A+B) into two fragments, TZJ2134-A (nt 7463-nt 9423) and TZJ2134-B (nt 9423-nt 11272), for phylogenetic and homology analyses. The results revealed that the homology between the two fragments and the corresponding parent viruses showed high similarity (Table 1). TZJ2134-A shared the highest nucleotide identity (99.17%) with the DV vaccine strain (Table 1) and belonged to DV-like isolates (Figure 2B). TZJ2134-B shared the highest nucleotide identity (99.73%) with the Amervac vaccine strain (Table 1) and belonged to Amervac-like isolates (Figure 2C). Both the DV and Amervac vaccine strains were PRRSV-1 MLV strains. To the best of our knowledge, only two reports from France and Denmark have described recombination events between two PRRSV-1 MLV strains (20, 31). One of them, PRRS-FR-2014-56-11-1, was the first recombinant strain derived from the Amervac vaccine strain and the DV vaccine strain described previously, with recombination events occurring at nt 500 to nt 1370, nt 3646 to nt 4272 and nt 4972 to nt 8430 in ORF1, as determined using RDP4 (31). Homology analysis showed that TZJ2134-(A+B) has the highest nucleotide identity (97.6%) with PRRS-FR-2014-56-11-1. PRRS-FR-2014-56-11-1 and TZJ2134-(A+B) are intermediates between Amervac-like isolates and DV-like isolates with DK-2011-05-23-9 and OLot/91 strains in the phylogenetic tree (Figure 1B). The recombinant and phylogenetic analysis results showed that all three viruses were recombinant strains derived from the Amervac vaccine strain and DV vaccine strain but with different recombinant patterns (Supplementary Figure S1) and formed a novel subgroup (DV+Amervac-like isolates) in the phylogenetic tree (Figure 1B).

In the late 1990s, PRRSV-1 MLVs were usually used to control PRRSV-1 infection in Europe (18). PRRSV-1 MLVs used worldwide include Porcilis PRRS (Merck), Amervac PRRS (Laboratories Hipra S.A.), ReproCyc PRRS EU (Boehringer Ingelheim), Pyrsvac-183 (SYVA Laboratories), and Ingelvac PRRSFLEX® EU (Boehringer Ingelheim) (32). They are used in Vietnam, Korea, Russia and Denmark and Taiwan in China. However, PRRSV-1 MLVs are not approved for use in mainland China. In this study we found a strain that is a product of recombination between vaccine strains (Amervac vaccine strain with the DV vaccine strain) and that these vaccines are not approved for use in China.

Since China is the largest pork importer in the world, the swine industry in China is vulnerable to the influence of the foreign swine industry. The existence of the TZJ2134 strain is a reminder that the swine surveillance at the Chinese customs should be strengthened.

## Conclusion

In summary, this study reports the first recombination event of the Amervac vaccine strain with the DV vaccine strain in China. These findings suggest that the surveillance against the introduction of foreign animal diseases should be strengthened.

## Data availability statement

The datasets presented in this study can be found in online repositories. The names of the repository/repositories and accession number(s) can be found at: www.ncbi.nlm.nih.gov/nuccore/, ON983961, ON974983.

## Ethics statement

The animal study was reviewed and approved by the Animal Ethics Committee of the School of Harbin Veterinary Research Institute of the Chinese Academy of Agricultural Sciences. Sampling procedures were performed in accordance with the guidelines of said committee The Animal Ethics Committee Approval Number was SYXK(Hei) 2011022.

## Author contributions

Conceived and designed the experiments: Z-JT and HZ. Performed the experiments: QS and HX. Contributed reagents or materials and assisted in some experiments: ZL, CL, and BG. Analyzed the data: QS, HX, and HZ. Contributed to the writing of the manuscript: QS, HX, CL, BG, HZ, and Z-JT. All authors contributed to the article and approved the submitted version.

## Funding

This study was supported by grants from the National Natural Science Foundation of China (Grant Nos. 32002315 and 32172890) and the China Postdoctoral Fund (Grant No. 2020M680788).

## Conflict of interest

The authors declare that the research was conducted in the absence of any commercial or financial relationships that could be construed as a potential conflict of interest.

## Publisher's note

All claims expressed in this article are solely those of the authors and do not necessarily represent those of their affiliated organizations, or those of the publisher, the editors and the reviewers. Any product that may be evaluated in this article, or claim that may be made by its manufacturer, is not guaranteed or endorsed by the publisher.
