# Supplementary material for: Emergence of a novel PRRSV-1 strain in mainland China: A recombinant strain derived from the two commercial modified live viruses Amervac and DV
Source: Front Vet Sci. 2022 Sep 9;9:974743. doi: 10.3389/fvets.2022.974743 (PMC9505512; doi:10.3389/fvets.2022.974743)
Supplement: Supplementary file 1 [file Table_1.DOCX]

Table S1 Primers used for detection and amplification of PRRSV-1

| Name | Primer sequence (5’-3’) | Position in genome | Product size (bp) |
| --- | --- | --- | --- |
| L12^a^ | CTTGTCCTAACGCCAAGTACC  CCAAAAGGGCCATGGCCTGTT | 1672-2112 | 441 |
| Ly-A^b^ | ATGATGTGTAGGGTATTCCCCC  GTCCAGAATTCCTGAGGAGGTG | 1-1936 | 1936 |
| Ly-B^b^ | CCTAGCGTCTGCTTACAGACTACC  AACGCCCCTGGGACACCACATA | 1847-4035 | 2189 |
| Ly-C^b^ | CAGCGCCAACTTTGGGAACCTG  CACAAAAGTTGAACGGTCGAGA | 3861-5923 | 2063 |
| Ly-D^b^ | TTGGTTCTGGTCTTGTGACAAC  TGGATTATTTGCTTGGATAACTC | 5698-7764 | 2067 |
| Ly-E^b^ | GTGGAGGTAAAGAAATCAACTGA  AGCCACCTTCACCATGTTTAT | 7523-9727 | 2205 |
| Ly-F^b^ | GGAGGTACCAGTCCCGTCGAGG  GGCTGTTGCCGGTCCTATACAC | 9591-11410 | 1820 |
| Ly-G^b^ | AGTTGGAAGGGCTCACGTGGTC  AGGCGAACGCCTCAGAAACC | 11304-12997 | 1694 |
| Ly-H^b^ | TATTATCACCACCAAATAGACGG  TTAATTTCGGTCACATGGTTC | 12887-15082 | 2196 |

a: detection primers;

b: primers used for complete genome amplification; Primers used for successful amplification are marked in red.
