# Supplementary material for: Emergence of a novel PRRSV-1 strain in mainland China: A recombinant strain derived from the two commercial modified live viruses Amervac and DV
Source: Front Vet Sci. 2022 Sep 9;9:974743. doi: 10.3389/fvets.2022.974743 (PMC9505512; doi:10.3389/fvets.2022.974743)
Supplement: Supplementary file 2 [file Table_2.DOCX]

Table S2 The reference sequence information of PRRSV-1

| **Strain** | **Area** | **Time** | **Accession no.** |
| --- | --- | --- | --- |
| **Strain** | **Area** | **Time** | **Accession no.** |
| Lelystad Virus | Netherlands | 1993 | [M96262](https://www.ncbi.nlm.nih.gov/nuccore/M96262) |
| BJEU06-1 | Beijing, China | 2006 | [GU047344](https://www.ncbi.nlm.nih.gov/nuccore/GU047344) |
| NMEU09-1 | Inner Mongolia, China | 2009 | [GU047345](https://www.ncbi.nlm.nih.gov/nuccore/GU047345) |
| SHE | Shanghai, China | 2009 | [GQ461593](https://www.ncbi.nlm.nih.gov/nuccore/GQ461593) |
| NVDC-NM1 | Inner Mongolia, China | 2011 | [JX187609](https://www.ncbi.nlm.nih.gov/nuccore/JX187609) |
| NVDC-NM2 | Inner Mongolia, China | 2011 | [KC492504](https://www.ncbi.nlm.nih.gov/nuccore/KC492504) |
| NVDC-NM3 | Inner Mongolia, China | 2011 | [KC492505](https://www.ncbi.nlm.nih.gov/nuccore/KC492505) |
| NVDC-FJ | Fujian, China | 2011 | [KC492506](https://www.ncbi.nlm.nih.gov/nuccore/KC492506) |
| FJEU13 | Fujian, China | 2013 | [KP860912](https://www.ncbi.nlm.nih.gov/nuccore/KP860912) |
| FJQEU14 | Fujian, China | 2014 | [KP860913](https://www.ncbi.nlm.nih.gov/nuccore/KP860913) |
| GZ11-G1 | Guizhou, China | 2011 | [KF001144](https://www.ncbi.nlm.nih.gov/nuccore/KF001144) |
| LNEU12 | Liaoning, China | 2012 | [KM196101](https://www.ncbi.nlm.nih.gov/nuccore/KM196101) |
| HKEU16 | Hong Kong, China | 2007 | [EU076704](https://www.ncbi.nlm.nih.gov/nuccore/EU076704) |
| HK3 | Hong Kong, China | 2003 | [KF287129](https://www.ncbi.nlm.nih.gov/nuccore/KF287129) |
| HK5 | Hong Kong, China | 2004 | [KF287130](https://www.ncbi.nlm.nih.gov/nuccore/KF287130) |
| HK10 | Hong Kong, China | 2004 | [KF287131](https://www.ncbi.nlm.nih.gov/nuccore/KF287131) |
| LV4.2.1 | Netherlands | 2004 | [AY588319](https://www.ncbi.nlm.nih.gov/nuccore/AY588319) |
| EuroPRRSV | USA, | 1999 | [AY366525](https://www.ncbi.nlm.nih.gov/nuccore/AY366525) |
| Amervac | Spain,Vaccine | [uncertain](javascript:;) | [GU067771](https://www.ncbi.nlm.nih.gov/nuccore/GU067771) |
| Lena | Belarus, | 2007 | [JF802085](https://www.ncbi.nlm.nih.gov/nuccore/JF802085) |
| MLV-DV | Spain | 1999 | KJ127878 |
| DV | Netherlands | 1996 | KF991509 |
| DK-2012-01-05-2 | Denmark | 2012 | KC862574 |
| DK-2010-10-10-3 | Denmark | 2010 | KC862568 |
| DK-2011-05-11-14 | Denmark | 2011 | KC862567 |
| DK-2011-05-23-9 | Denmark | 2011 | KC862569 |
| Cresa3262 | Spain | 1992 | JF276431 |
| 01CB1 | Thailand | 2001 | DQ864705 |
| 14432/2011 | Hungary | 2011 | KR296711 |
| DK-2008-10-5-2 | Denmark | 2008 | KC862573 |
| SD01-08 | USA | 2001 | DQ489311 |
| SD03-15_P83 | USA | 2003 | KU131560 |
| PRRS-FR-2005-29-24-1 | France | 2005 | KY366411 |
| 195-05 | UK | 2005 | KU560579 |
| Cresa3249 | Spain | 2005 | JF276433 |
| Cresa3256 | Spain | 2005 | JF276432 |
| Olot/91 | Spain | 1991 | KF203132 |
| 9625/2012 | Hungary | 2012 | KJ415276 |
| Cresa3267 | Spain | 2006 | JF276435 |
| ESP-1991-Olot91 | Denmark | 1991 | KC862570 |
| CReSA228 | Spain | 2013 | KX249755 |
| HK8 | Hong Kong, China | 2004 | KF287128 |
| 94881 | USA | 2006 | KT988004 |
| IVI-1173 | Switzerland | 2012 | KX622783 |
| AUT14-440 | Austria | 2014 | KT334375 |
| E38 | South Korea | 2007 | KT033457 |
| KNU-07 | South Korea | 2007 | FJ349261 |
| GER09-613 | Germany | 2013 | KT344816 |
| AUT13-883 | Austria | 2013 | KT326148 |
| CReSA17 | Spain | 2014 | KX249749 |
| CReSA38 | Spain | 2014 | KX249750 |
| CReSA3 | Spain | 2013 | KX249748 |
| CReSA70 | Spain | 2014 | KX249752 |
| CReSA46 | Spain | 2014 | KX249751 |
| CReSA100 | Spain | 2014 | KX249753 |
| CReSA261 | Spain | 2013 | KX249756 |
| DK-2003-6-5 | Denmark | 2003 | KC862571 |
| DK-2003-7-2 | Denmark | 2003 | KC862572 |
| DK-1992-PRRS-111 92 | Denmark | 1992 | KC862566 |
| 07V063 | Belgium | 2007 | GU737264 |
| 13V117 | Belgium | 2013 | KT159249 |
| 13V091 | Belgium | 2013 | KT159248 |
| SU1-Bel | Belarus | 2010 | KP889243 |
| PRRS-FR-2014-56-11-1 | France | 2014 | KY767026 |
