# Supplementary material for: Emergence of a novel PRRSV-1 strain in mainland China: A recombinant strain derived from the two commercial modified live viruses Amervac and DV
Source: Front Vet Sci. 2022 Sep 9;9:974743. doi: 10.3389/fvets.2022.974743 (PMC9505512; doi:10.3389/fvets.2022.974743)
Supplement: Supplementary file 3 [file Image_1.pdf]

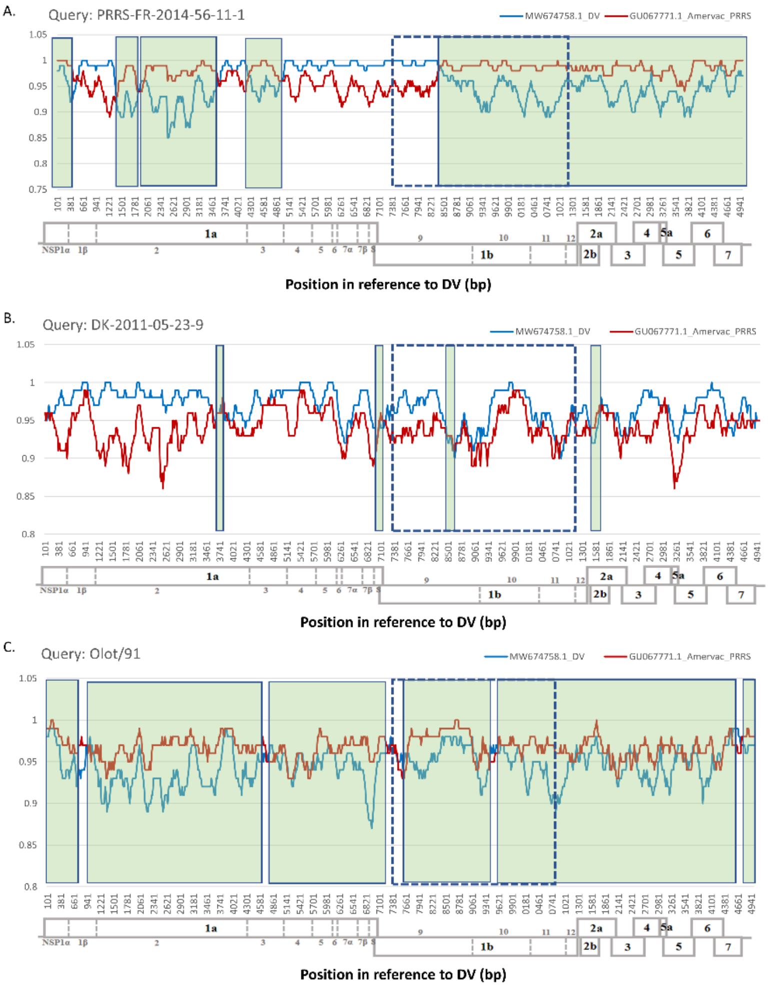

FIGURE S1 Recombination analysis of DV+Amervac-like isolates. (A) Recombination analysis of PRRS-FR-2014-56-11-1. (B) Recombination analysis of DK-2011-05-23-9. (C) Recombination analysis of OLot/91. Similarity comparisons were performed using PRRS-FR-2014-56-11-1, DK-2011-05-23-9 and OLot/91 as the query sequences and DV (blue) or Amervac (red) as the parent strains. The recombination regions are shown in green. The position of TZJ2134-(A+B) in the whole genome is shown in the blue dotted box.
